# Detection of surface enhanced Raman scattering active hotspot using near field scanning optical microscopy

**DOI:** 10.1038/s41598-024-61503-7

**Published:** 2024-05-08

**Authors:** Mohammad Kamal Hossain

**Affiliations:** 1 Interdisciplinary Research Center for Sustainable Energy Systems (IRC-SES), Research Institute, King Fahd University of Petroleum & Minerals (KFUPM), 31261 Dhahran, Saudi Arabia; 2 K.A.CARE Energy Research & Innovation Center at Dhahran, King Fahd University of Petroleum & Minerals (KFUPM), 31261 Dhahran, Saudi Arabia

**Keywords:** Near-field spectroscopy, Surface-enhanced Raman scattering, Scanning near-field optical microscope, Hotspot, Finite-difference time-domain analysis, Nanoscience and technology, Optics and photonics

## Abstract

Hotspots are high-intensity electromagnetic zones that form, for example, at the interstitials of plasmonic nanoaggregates, resulting in a considerable rise in the enhancement factor. However, it is inevitable to achieve specific nanometric geometry as well as a suitable technique to capture the details of hotspots. We report near-field surface-enhanced Raman scattering (SERS) spectroscopy of a well-defined gold nanoaggregate of a few nanoparticles adsorbed with a small number of target analytes. A spectrally and spatially resolved SERS measurement setup using an aperture near-field scanning optical microscope (a-NSOM) facilitated the direct observation of localized electromagnetic (EM) fields at the interstitials through SERS. Correlated optical image and corresponding nanometric geometry were captured through the home-built a-NSOM setup. Near-field SERS spectra were recorded at different sites of interest. It was evident that the interstitial positioned at the center of the tetramer provided the most intense Raman scattering, implying the possibility of a SERS-active hotspot therein. SERS bands of the spectrum of the Raman-active dye Rhodamine 6G recorded at the same hotspot coincided well with those reported so far. It was noteworthy that most of the SERS bands in such scenery got enhanced. Such direct observation with high spatial resolution is indispensable to understanding the origin of localized EM fields at “hotspots” and the EM enhancement factor in the SERS process. A finite-difference time-domain (FDTD) analysis was carried out to validate the results.

## Introduction

Surface-enhanced Raman scattering (SERS) is an analytical spectroscopy technique that has generated significant interest in a variety of research fields, including but not limited to oncology, chemical analysis, food security and quality, environmental issues, and energy^1–5^. In SERS, weak Raman signals (approximately one out of a million photons) are enhanced by up to several orders of magnitude. Two mechanisms are acknowledged to be mainly responsible for giant enhancement in SERS: one, electromagnetic (EM) enhancement due to localized EM field distribution at the “hotspots”, and two, chemical enhancement due to charge transfer between target analyte and SERS-active electrode^1,6–10^. It is noteworthy that in SERS enhancement, the EM enhancement effect through the “hotspot” mechanism is recognized to be several orders higher than that of chemical enhancement. “Hotspot” or “active site” is defined as the site of interest where a strong and localized electromagnetic (EM) field happens to exist and thus contributes to giant SERS enhancement^6,8,10,11^. Although the interstitial of two adjacent nanoparticles was reported to be the best hotspot, strong EM near-fields can be induced at asperities, such as the apexes of triangles, corners of cubes, or edges of arbitrary shapes^12–17^. It is well-demonstrated that such confined and strong near-field distributions are mainly due to localized surface plasmon resonances (LSPRs), which are defined as a collective and coherent oscillation of conduction electrons^1,6–8,18,19^.

Although hotspots were theoretically demonstrated decades ago, recent experiments using scanning near-field optical microscopy, photoemission electron microscopy, direct mapping of the local optical density of states, and second harmonic generation have allowed for the experimental realization of such intense EM near-field sites^12,20–23^. Due to the nature of strong electromagnetic fields, the confinements in gap-mode plasmonics are considered firmly constrained inside a nanometric volume of hotspot. In such cases, an infinitesimal change in the nanometric geometry will create new pathways for the contained energy to percolate through the coupling of surrounding hotspots and thus change the distributions of the electromagnetic near-field. However, there are consistence challenges to understanding the microscopic correlation between localized EM near-field and confined optical fields. Since the dimension of the hotspot (roughly 1–10 nm) is well below the optical diffraction limit, characterizing hotspots has proven to be a challenging experimental task^24,25^. As a result, the resolution of SERS measurements linked to EM hotspots is limited to the 200–400 nm length scale, which is significantly larger than the hotspots’ actual relevant length scale. One of the major challenges in capturing SERS-active hotspots is achieving reproducibility in the fabrication of SERS substrates. SERS-active hotspots require precise control over the size, shape, and distribution of the nanoscale features, which can be difficult to achieve using conventional fabrication techniques. This can lead to variability in the SERS response, making it difficult to reliably capture the hotspots necessary for high-sensitivity detection.

In the field of optical microscopy, nanoscale imaging of optical confinement has been a long-standing desire and persistent difficulty^12,26,27^. Spectrally- and spatially-resolved measurements, particularly correlated observations of such hotspots, have been seldom reported and remain a difficult task primarily due to the diffraction limit, a fundamental limit on the maximum resolution of an optical image^28^. Based on the diffraction theory of light, the possible spatial resolution of the collected image was calculated to indicate the maximum possible spatial resolution of hundreds of nm in the visible and up to several microns in the infrared^29^. For understanding and interpreting near-field optics, which is frequently governed by nanoplasmonics and nanophononics, such resolution is not good enough. In the early 1990s, Dieter Pohl and Aaron Lewis developed near-field scanning optical microscopy (NSOM), a cutting-edge microscopic technique that demonstrated an unprecedented ability to surpass this diffraction limit. By sweeping the NSOM probe or tip across the target surface and collecting or making it easier to extract emitted or scattered photons, NSOM supports the ability to carry out spectrally- and spatially-resolved measurements. Near-field tips, or NSOM probes, primarily modify or improve the incident optical field locally inside the nanometric geometry. Any surface-sensitive optical process, such as SERS, will be significantly impacted by such a scenario. The detail of the NSOM technique has been demonstrated elsewhere in several reviews^30–34^. On the other hand, hotspots in SERS are so important that just a small amount of target analytes adsorbed at the hotspots generates the maximum portion of the SERS signal, which is crucial in trace analysis and single-molecule identification^35–37^. Therefore, to understand the inherent feature of an analyte at the hotspot, it is highly appreciated to capture the phenomenon through a spectrally- and spatially-resolved experimental setup. Many spectroscopic studies of SERS hotspots made up of either randomly arranged structures or nanofabricated plasmonic antennas have been conducted in recent years^38–42^. Hotspots have been extensively investigated for particle and molecule entrapment, improved photochemistry, and even nanolithography in addition to chemical analysis using SERS^42^. Snapshots of hotspots have been obtained by near-field scanning optical microscopy with a resolution of about 10 nm^43,44^. Scanning (transmission) electron microscopes have unveiled images with a resolution of more than 10 nm utilizing electron energy-loss spectroscopy^45^. Interestingly, one could find that most of the single-molecule SERS studies concentrated on the performance of SERS-active substrates, avoiding qualitative and quantitative measures of SERS hotspots due to the limitations of the experimental setup. Simultaneous spectrally- and spatially-resolved SERS measurements provide rich information about the SERS hotspot, including trace detection of target analytes and static and dynamic characteristics of the same, apart from nanogeometry-, wavelength- and polarization-dependent characteristics of the hotspot itself^12,15,18,28,46–48^.

As this study has shown, correlated measurements are important for many applications in nanoscience and technology. For instance, the non-destructive, label-free SERS measurements offer fingerprint structural information on molecules. Researchers can get more thorough data and insight by linking SERS with topographical measurements, as this paper explains. Correlated data, including high-resolution photos, three-dimensional structures, site-specific spectral data, and indications of likely mechanisms involved, may be obtained thanks to this integration. Additionally, advances in nanoscopy are facilitated by a flexible setup like the a-NSOM employed in this work. The resolution constraints of conventional optical microscopy are overcome by NSOM. Conventional optical microscopy has a limit of about 200 nm, but NSOM allows for optical resolution of less than 50 nm. To some extent, the study presented in this paper provides materials experimentalists with new opportunities to perform correlated measurements more reliably and efficiently. Correlated SERS and topography data are known to improve the analysis’s accuracy and dependability. Correlating spectroscopic results with scanning electron microscopy (SEM) images is crucial, though, especially when using SERS substrates with nonspherical symmetry as these are utilized in this work. This association makes it possible to better grasp optometrology and nanogeometry at the same location.

In this work, a-NSOM was used to capture snapshots of hotspots at the interstitial through SERS measurements. A fairly good correlation between topography and optical images was observed. Near-field SERS spectra were recorded at five sites of interest. It was evident that the interstitial positioned at the center of the tetramer provided the most intense Raman scattering, implying the possibility of a SERS hotspot therein. SERS bands of the spectrum of the Raman-active dye Rhodamine 6G recorded at the same hotspot coincided well with those reported so far. It was noteworthy that most of the SERS bands in such scenery got enhanced. Such direct observation with high spatial resolution is indispensable to understanding the origin of localized EM fields at “hotspots” and the EM enhancement factor in the SERS process. A finite-difference time-domain (FDTD) analysis was carried out to validate the results using an FDTD model very similar to the one under investigation.

## Materials and methods

### Home-built a-NSOM setup

**Figure 1:**
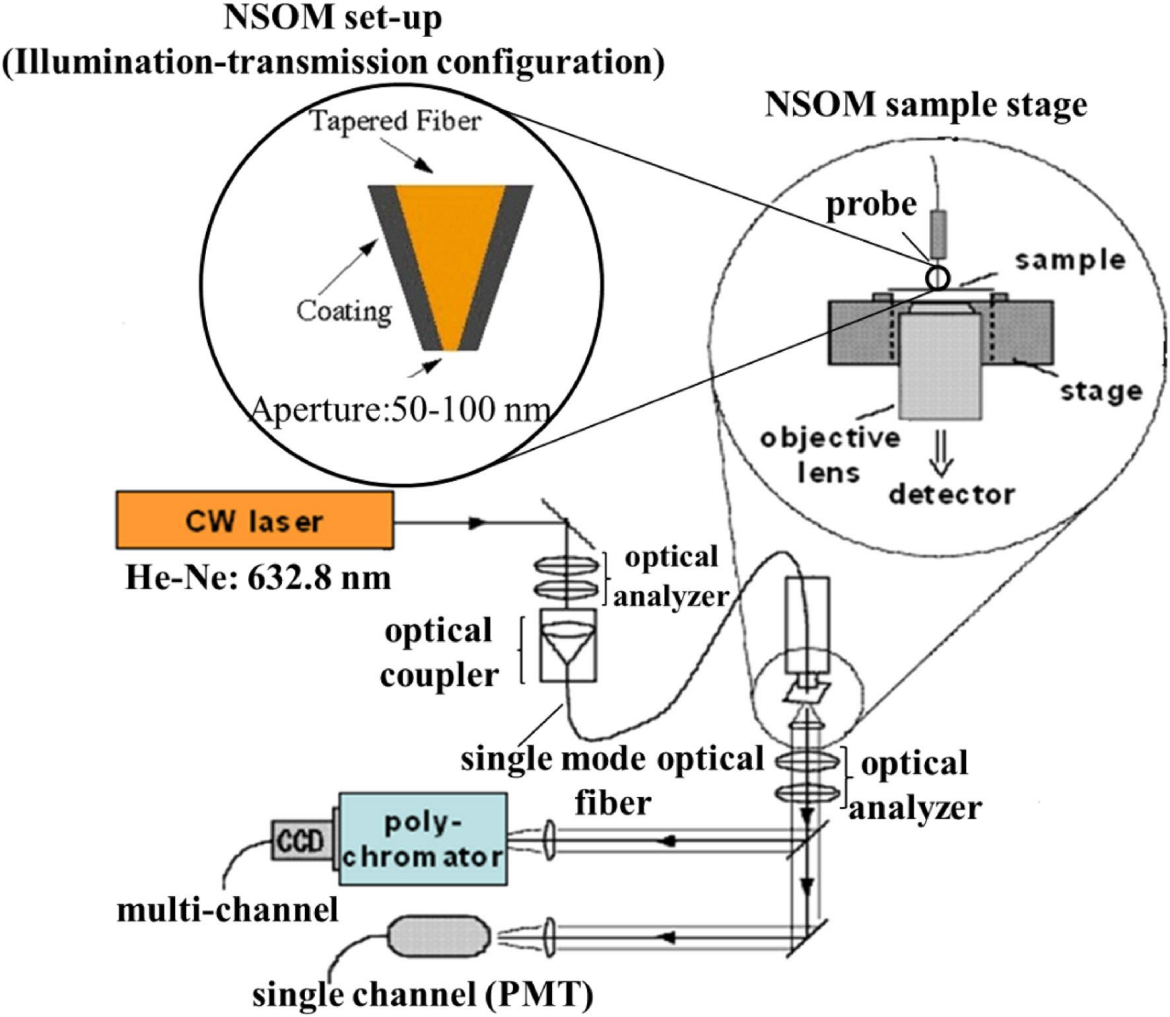
Free-hand schematic depicting the home-built a-NSOM facility used under this investigation. Insets (i) and (ii) represent the detailed configuration of the NSOM stage and tapered optical fiber tip used as a-NSOM probe.

The a-NSOM setup used in this study was built by combining three main sections, viz., excitation source, sample stage, and detection system, as shown in Fig. 1^12,18^. The home-build a-NSOM was consisted of a probe (from JASCO, NPU-102B, aperture 50–100 nm), a closed-looped x–y–z stage (from nPoint) and an objective lens (from Nikon, NA 0.85), leading to a CCD array detector (from Andor DV401-F1). All the a-NSOM measurements were carried out in illumination-transmission mode configuration. A He–Ne laser (632.8 nm) was used to excite Raman scattering from the sample. The laser was connected to a single-mode optical fiber through an optical coupler. Another end of the optical fiber was used as an NSOM probe. A gold-coated apertured near-field probe tip (aperture diameter 50–100 nm) was used to localize the excitation, and the measurements were carried out in illumination-transmission configuration under ambient conditions. The tip-to-specimen distance was kept as low as 10–15 nm by the tuning-fork feedback mechanism. The morphology of the sample surface was verified by shear-force topographic measurements captured simultaneously by the a-NSOM system. The scattered Raman photon was collected by an objective lens mounted below the sample stage and detected by a polychromator-CCD (for multichannel detection) and/or an avalanche photodiode (for single-channel detection). The home-built a-NSOM setup facilitated recording correlated metrology and topography simultaneously at the very same position without disturbing temporal and spatial resolution.

### Well-defined gold aggregate and near-field SERS

Achieving a monolayer and well-defined aggregate was the primary prerequisite to validating the hotspot mechanism in SERS, and therefore specific care has been taken in this context. A high-purity colloidal solution of gold nanoparticles suspended in water (mean diameter 98.9 nm with a mean range of 96.0 to 104.0 nm and a concentration of 5.6 × 10^9^ nanoparticles/mL) was purchased from British BioCell International (Cardiff, U.K.) and used as received. A glass substrate functionalized with trimethoxy-[3-(methylamino)propyl] silane was purchased from Matsunami and used as a substrate to immobilize gold nanoparticles atop. We employed a colloidal solution of gold nanoparticles with a limited size distribution. We utilized a unique self-assembly method, free of extra surfactant molecules or capping, to create the monolayered nanoaggregates of gold nanoparticles. Using a glass coverslip that had been treated with trimethoxy-[3-(methylamino)propyl] silane, we first dropped a colloidal solution of the gold nanospheres in this approach. The droplet was then covered by a slide glass that had certain spacers on it. Consequently, the arrangement of the two glass substrates stopped the gold nanoparticles from aggregating, resulting in the formation of the two-dimensional arrays of particles as well as a variety of N-particles nanoaggregates where N was 2 or more. The line profiles of AFM images revealed a monolayer feature (neither multilayer nor agglomerate), in which the step height of sample layer and particle size are equal. It is speculated that the van der Waals force and the electrostatic force should be balanced to decide the gap distance. Furthermore, we believe that controlling the convection flow throughout the droplet’s drying process is crucial. It was determined that there were gaps and that the constituent particles were not in direct touch from the SEM investigations. Using the method, we repeatedly created the nanoaggregates and verified that the gaps, which have an average spacing of few nanometers as elaborated in Supporting Information. A salient feature of N-particle gold nanoaggregates where N is small in number has been confirmed by scanning electron microscope (SEM, JEOL FE6500) and shear-force topographic measurements of the a-NSOM. It was revealed that the constituent nanoparticles were not in direct contact, and therefore the tiny gaps played a crucial role in generating SERS-active hotspots^12,18^.

Near-field SERS-activity in the presence of a well-defined gold nanoaggregate was carried out using a Raman-active dye, Rhodamine 6G (R6G, C_28_H_31_N_2_O_3_Cl). R6G was used as received from Chroma GesellschaftSchmid GMBH & Co. without any further modification. The gold nanoaggregate immobilized on the coverslip was spin-coated with a water solution of R6G, and thus the analytes spread out due to strong centrifugal force. A small droplet of ca. 50–100 nL solution of R6G (1 × 10^–6^ M) was used in this case. It was speculated that the droplet dropped vertically on the rapidly spinning substrate would facilitate to adsorb small quantity of dyes. High-speed spin coating with such a small volume of diluted solution does not cause aggregations on the sample. The coverage of R6G molecules was estimated to be several hundred molecules per 100 nm × 100 nm considering the homogenous distribution of the solution. Near-field SERS measurements were carried out at ca. 40 nm steps across the scan area of 1 µm × 1 µm with 1 s of exposure time. The near-field SERS spectra and the mappings were analyzed by OriginPro 2023 (Copyright © 1991–2022; OriginLab Corporation) and SPIP (ver. 6.7.0; Image Metrology) respectively.

### FDTD simulation

In the SERS study, understanding EM near-field distributions is inevitable, as elaborated above in the introduction. In this context, a model system was designed in such a way that the geometry and parameters represented the nanoaggregate under investigation and the NSOM setup very closely. A close-pack and interacting tetramer along with two isolated nanoparticles very similar to those observed in morphological investigations were developed and simulated by PLANC-FDTD (Information and Mathematical Science Lab. Inc., Tokyo, Japan; Ver. 6.2; Copyright © 1994–2004). The PLANC-FDTD used as a source code for FDTD analysis is one of the primitive software. Electromagnetic near-field distributions along various planes for s-, p-, and 45° incident polarizations have been extracted and analyzed to correlate with those obtained in near-field SERS observations. Further details have been elaborated on in the later part of the text. In order to correlate the EM near-field distribution, a 632.8 nm excitation that is normal to the model geometries was utilized in the FDTD simulation. In order to keep things simple, nanoobjects were assumed to be smooth and spherical, despite the fact that the individual nanoparticles differed from one another, notably in size and form, as seen in topographical studies.

## Results and discussion

### Correlated optometrology: near-field SERS and shear-force topology

SERS is realized mainly due to the influence of strong EM near-field distribution (known as the EM enhancement mechanism) induced at the hotspot. Therefore, the characteristics of the hotspot as well as the incident field play a vital role in defining whether incident energy will get confined at the interstitial and thus energize the analyte positioned at the very same spot. The number of interstitials increases with the number of constituent nanoparticles, wherein EM near-field distributions get hybridized and coalesce with nearby distributions. The a-NSOM setup used in this current study gave us huge opportunity to study correlated optometrology using isolated nanosphere, dimer, trimer, tetramer and several more nanoaggregates^12,18^. As for hotsite, we acknowledge that every interstitial contributing to hotsite within the nanoaggregate is different from each other. It is to be mentioned that by NSOM, such sites of interest can be tapped, but it is quite impossible to tap the same hotspot region after dismounting the substrate from NSOM setup.

It is extremely challenging to capture the correlated hotspot topology and near-field optical confinement without the assistance of near-field microscopic investigations, as demonstrated below.

**Figure 2:**
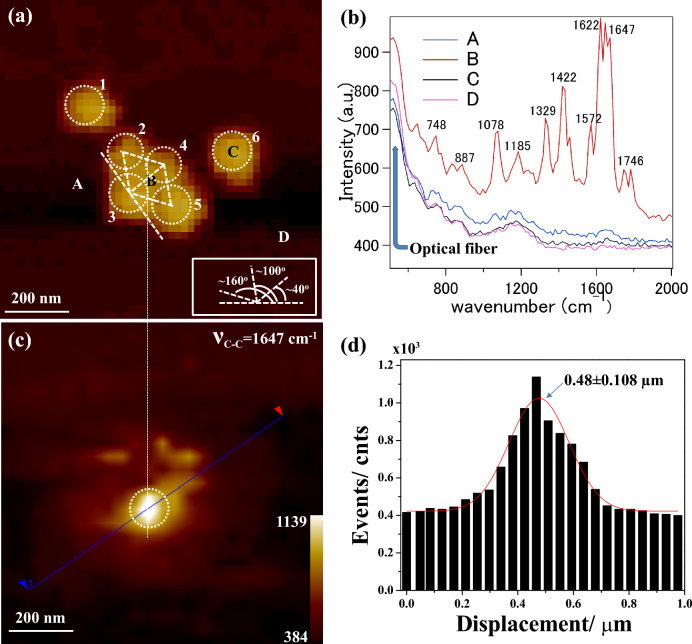
(**a**) Shear-force topography captured simultaneously during a-NSOM measurement; constituent nanoparticles are marked by “1”, “2”, “3”, “4”, “5” and “6” therein; inset: directions of interparticle axes with a reference. (**b**) Near-field SERS spectra extracted from four different sites of interest as marked by “A”, “B”, “C” and “D” in (**a**). (**c**) Near-field SERS mapping at the 1647 cm^−1^ SERS band of R6G; The white vertical dashed line guides the reader to follow the optical confinement that corresponded to the interstitial in (**a**) and (**d**) Bar graph of SERS photon count along the blue line in (**c**). Individual scale bars included in (**a,c**). The color bar in (**c**) represents the SERS intensity.

Figure 2a shows the shear-force topography of an Au nanoaggregate captured simultaneously during the near-field SERS measurements using an a-NSOM facility. An aggregate of six distinct nanoparticles was noted, wherein four nanoparticles marked as “2”, “3”, “4”, and “5” were closely interacting, making four interstitials along three different interparticle axes. Nanoparticles marked “1” and “6” were isolated. The directions of the interparticle axis between constituent nanoparticles were shown in the white rectangle of Fig. 2a. The interparticle axes between nanoparticles “2” and “3” and nanoparticles “4” and “5” were estimated to be ~ 100°, whereas those between nanoparticles “2” and “4” and nanoparticles “3” and “5” were along ~ 160°. The interparticle axis between nanoparticles “3” and “4” was estimated to be ~ 40°. It is well-acknowledged that the strongest EM near-field distribution is achieved at the perfect match between the interparticle axis and incident polarization due to constructive plasmon coupling. Detailed EM near-field distributions through FDTD simulation have been amended in the later part of the text. However, the direction of the interparticle axis is very critical to elucidate the SERS enhancement at a particular hotspot. Interestingly, a snapshot of such a hotspot through correlated optometrology is inevitable to understand the SERS characteristics. As elaborated in the experimental section, the entire specimen surface was scanned by the near-field probe of the a-NSOM, and SERS spectra were collected from each pixel of the mapping. Figure 2b represents near-field SERS spectra of R6G recorded at four specific sites of interest as marked by “A” (nearby the core aggregate), “B” (at the interstitial between nanoparticles “3” and “4”), “C” (at the monomer marked as “6”), and “D” (far from the core aggregate). It was evident that near-field SERS of R6G were strongly enhanced at “B” compared to those observed at “A”, “C” and “D”. A few near-field SERS bands of R6G at 748, 887, 1078, 1187, 1329, 1422, 1572, 1622, 1647, and 1746 cm^−1^ were observed and mentioned therein in Fig. 2b. The abovementioned near-field SERS bands coincided well with the reported work. It is noteworthy that several unknown or shifted near-field SERS bands of R6G were recorded in this instant. Further detailed near-field SERS bands, band assignments, and the corresponding mappings have been demonstrated in the later part of the text. Figure 2c displays a typical near-field SERS mapping obtained at the near-field SERS band of 1647 cm^−1^ (aromatic C–C stretching mode) of R6G. A vertical white dashed line across Fig. 2a,c guided the reader to correlate shear-force topology and the corresponding near-field SERS mapping. Intensity distributions of the mapping revealed that intense near-field SERS was observed at the interstitial of nanoparticles “3” and “4”. The intensities of the near-field SERS band 1647 cm^−1^ of R6G (aromatic C–C stretching mode) along the solid blue line as shown in Fig. 2c were plotted in Fig. 2d along with a Gaussian fit in red. It was noteworthy that the full width at half maximum (FWHM = 2 × 0.108 µm = 0.216 µm) of the maximum near-field SERS intensity was spread over ± 108 nm, as shown in Fig. 2d. There were several sites of intense near-field SERS apart from the strongest one, as shown in Fig. 2c. The spectral characteristics of those sites were analyzed further and stated below.

**Figure 3:**
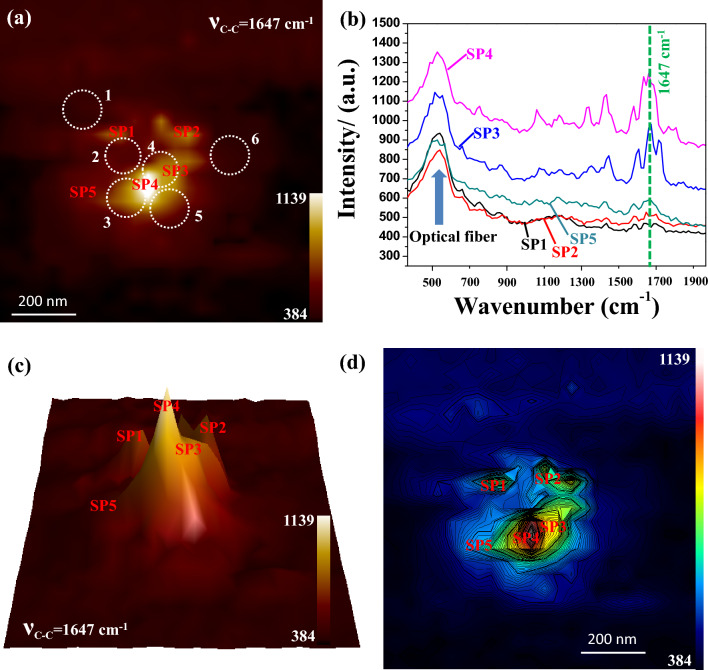
(**a**) Near-field SERS mapping at the 1647 cm^−1^ SERS band of R6G overlapped with possible positioning of constituent nanoparticles marked by the white dotted circles therein; Five sites of interest are marked by “SP1”, “SP2”, “SP3”, “SP4” and “SP5”. (**b**) Near-field SERS spectra obtained at five sites of interest as marked in (**a**); Vertical green dotted line indicates 1647 cm^−1^ band, (**c**) 3D hawk-eye view of the same near-field SERS images as shown in (**a**) and (**d**) contour plot of the same near-field SERS images as shown in (**c**). Individual scale bars included in (**a,d**). The color bar in (**c**) represents the SERS intensity.

Figure 3a shows the same typical near-field SERS mapping (1647 cm^−1^, aromatic C–C stretching mode of R6G) overlapped with possible constitute nanoparticles. Out of many intense near-field SERS sites, five sites of interest were demonstrated as marked by “SP1” (at the top of nanoparticle “2”), “SP2” (in between nanoparticles “4” and “6”), “SP3” (next to nanoparticle “4”), “SP4” (at the interstitial of nanoparticles “4” and “3”) and “SP5” (to the left of nanoparticle “3”) as shown in Fig. 3a. The near-field SERS spectra of R6G recorded at abovementioned five sites interest are shown in Fig. 3b. At “SP4”, most of the SERS bands found strongly enhanced in the near-field SERS spectrum of R6G. Some of the SERS bands were found coincided well with reported works as explained earlier, whereas others were theoretically predicted. All the near-field SERS bands and corresponding band assignments were tabulated and elucidated in details in the later part of the text.

To the extend, near-field SERS mapping for individual bands of R6G were extracted and elaborated. At “SP3”, some of the bands of near-field SERS spectrum of R6G were found enhanced. However, at “SP1, SP2” and “SP5”, the bands intensities of near-field SERS spectrum of R6G were found to be almost negligible compared to those obtained at “SP4” and “SP3”. The site “SP4” represents the strongest site of near-field SERS enhancement, whereas the same site corresponded to the interstitial of constituent nanoparticles “3” and “4” as shown in Fig. 3a. As stated earlier in experimental section, the incident laser was having polarization angle of 41° that was closely matched with the interparticle axis of constituent nanoparticles “3” and “4”. Therefore, the interstitial between nanoparticles “3” and “4” was in fact the hotspot; a site of strongly localized plasmon coupling. On the other hand, the site “SP3” was found closely located to the site “SP4” and thus strong near-field SERS of R6G was observed. A 3D hawk-eye view of the same near-field SERS mapping (1647 cm^-1^, aromatic C–C stretching mode of R6G) was shown in Fig. 3c. The strongest SERS enhancement was noted at site “SP4” indicating highest peak, whereas the one next to the strongest enhancement was found at the shoulder. A contour plot of the near-field SERS intensity (1647 cm^-1^, aromatic C–C stretching mode of R6G) overlapped with near-field SERS mapping was shown in Fig. 3d. The contour plot reconfirmed that the strongest near-field SERS enhancement was observed at site “SP4”. It is well-acknowledged that the signal enhancement of a particular SERS band depends on the interaction characteristics of the “plasmonic surface-analyte” system. The position and orientation of analyte with respect to localized EM near-field distribution are crucial to determine the characteristics of SERS band. The SERS bands that were properly aligned with the plasmon-enhanced electromagnetic field were predicted to be amplified, but the ones that were not aligned fell short of increasing the signals. Furthermore, the SERS bands that are amplified are those nearest to the plasmon-active surface, resulting in non-reproducible spectra from the interaction between the substrate and the analyte, which has been one of the hurdles in the widespread application of SERS. The SERS spectrum at point B in Fig. 2b is closely similar to that obtained at SP4 in Fig. 3. In SERS spectrum at point B in Fig. 2b, 10 SERS bands with strong intensities were marked, although there were another 4 SERS bands with lower intensities. All these SERS bands of R6G were tabulated along with possible band assignments in Table 1. Most of the SERS bands coincided well with reported work whereas some bands were predicted in theoretical study as mentioned in Table 1. As shown in Fig. 2b, the relative intensity of 1647 cm^−1^ SERS band (C–C stretching in xanthene ring) of R6G at the point B was recorded to be ~ 350 cnts. On the other hand, the relative intensities of 1647 cm^−1^ SERS band of R6G at the point SP4, SP3, SP5, SP2 and SP1 in Fig. 3b were recorded to be ~ 320, ~ 290, ~ 190, ~ 50 and ~ 40 cnts respectively. SERS band at 1647 cm^−1^ (C–C stretching in xanthene ring) of R6G has been identified and marked in Fig. 3b.

**Table 1:** SERS bands and corresponding band assignments of R6G obtained under this investigation as well as those obtained experimentally and theoretically.

Mode of vibration (cm^−1^)	Experimental	Hayazawa et al.^49^	Theoretical^50^
	1746		1716
C–C stretching in xanthene ring	1647	1647	1652
hybrid mode (phenyl ring with COOC2H5	1622		1615
		1596	
C–C stretching in phenyl ring	1572	1570	1577
C–N stretching in NHC2H5	1458		1458
		1532	
	1422		1419
		1503	
C–C stretching in xanthene ring	1329	1359	1351
		1308	
	1238	1269	
C–H in-plane bending in xanthene ring	1185	1185	1192
		1120	
	1078	1084	1084
	887	919	895
	832		819
C–C op bend	748	766	771
	649		631
		608	

### Near-field SERS: selective bands validated by experiment and DFT calculation

**Figure 4:**
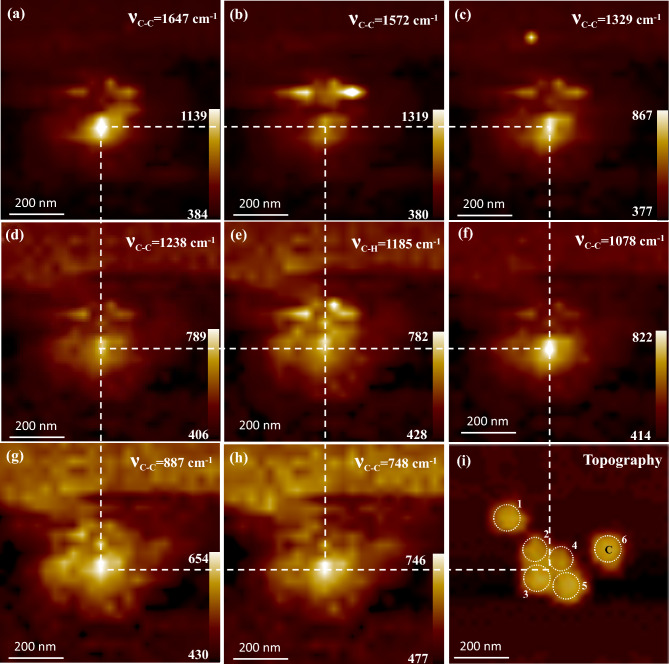
(**a–h**) Near-field SERS mapping at 1647, 1572, 1329, 1238, 1185, 1078, 887, and 748 cm^−1^ SERS bands of R6G, respectively, and (**i**) shear-force topography of the nanoaggregate along possible 6 nanoparticles. Individual scale bars included. The color bar represents the SERS intensity.

**Figure 5:**
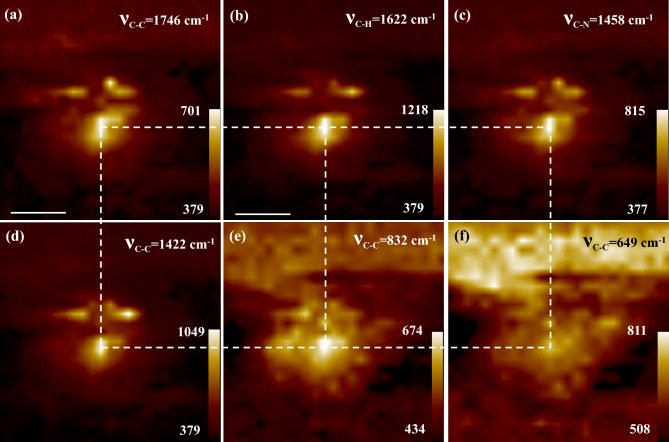
(**a–f**) Near-field SERS mapping at 1746, 1622, 1458, 1422, 832, and 649 cm^−1^ SERS bands of R6G, respectively. Individual scale bars included. The color bar represents the SERS intensity.

It was explained earlier that some of the bands of near-field SERS of R6G were found enhanced at specific sites, whereas at other sites, some bands were found weak. The spectrum of near-field SERS is mostly similar to the spectrum of far-field SERS, with the exception that the EM near-field is very intense in the near-field scenario due to the effect of the evanescent field. In addition to EM near-field, the orientation of the analyte also influences the intensity of a particular near-field SERS band. Some specific and selective modes of enhancement of R6G molecules were observed, which had never been reported even by single-molecule SERS detection, with an enhancement factor of upto 10^15^. The observed Raman shifts were in good agreement, partially with the simulated values, considering the tip-enhancement effect^50,51^. The SERS bands found in this experiment are summarized in Table 1, along with band assignments and comparisons with those reported experimentally and theoretically. Hayazawa et al. reported^49^ that many selective modes of enhancement of R6G molecules were possible by tip-enhanced experimental setup in the near field, such as near-field SERS by a-NSOM used in this current investigation. As shown above in Fig. 3b, the near-field SERS spectrum of R6G at the right hotspot “SP4” indicated possible bands located at 1746, 1647, 1622, 1572, 1458, 1422, 1329, 1238, 1185, 1078, 887, 832, 748, and 649 cm^−1^ wavenumbers. Some of these SERS bands, such as bands at 1647, 1572, 1329, 1238, 1185, 1078, 887, and 748 cm^−1^ wavenumbers, coincided well with those reported experimentally by Hayazawa and the group, although a bit shifted peaks are well-acknowledged in near-field SERS measurements^49^. On the other hand, all the SERS bands observed under this investigation were supported by the density functional theory reported theoretically by Watanabe and the group^50^. Further to clarify the correlation between near-field SERS-active sites and nanoscale topography, a near-field SERS mapping was extracted for the abovementioned SERS bands of R6G, as shown in Figs. 4 and 5.

Figure 4 shows near-field SERS mappings for the selective SERS bands of R6G observed under this study, which coincided well with the experimentally reported works. Figure 4a–h represent near-field SERS mappings of R6G extracted for the bands located at 1647, 1572, 1329, 1238, 1185, 1078, 887, and 748 cm^−1^ wavenumbers, respectively. Figure 4i displays the shear-force topography of the same area captured simultaneously during near-field SERS measurements. The dashed squares, as mentioned in Fig. 4 guide the reader to follow the site of the strongest near-field SERS intensity for each individual band. It is noteworthy that the vertices of the four squares are pointing at the strongest sites, and all the strongest sites matched well to the interstitial “SP4” in the topography, as shown in Fig. 4i. As stated earlier in the preceding section, interstitial “SP4” facilitated localization of strong EM near-field and thus a strong enhancement in SERS characteristics. It is to be noted that the SERS bands at 1572 and 1238 cm^*−*1^ that represent C–C stretching in phenyl ring and C–C stretching in xanthene ring respectively were found to have higher intensities outside the hotspot. It is speculated that these two vibrational bands were not enhanced or activated at the hotspot in this particular study. In SERS, hotspots are highly localized regions of intense local field enhancement. Hotspots play a crucial role in enhancing the SERS signal of analyte adsorbed on or near the surface of plasmonic nanoaggregates. However, it is important to note that the enhancement of specific SERS bands depends on various factors, including the excitation wavelength, the molecular orientation, background emission and the interaction between the analyte and the plasmonic nanoaggregates. We acknowledge that the SERS mapping at 1572 cm^−1^ band (C–C stretching in xanthene ring) of R6G as shown in Fig. 4b indicates higher intensity at the region outside the hosite and even outside the single nanoparticle. One could note from the SERS mapping that the intensity of the 1572 cm^−1^ band was comparable to the maximum intensity of the 1647 cm^−1^ band in two different locations, not at the same location. The intensity distribution in the SERS mapping at the 1572 cm^−1^ band was not evenly distributed as shown in Fig. 4b. This observation could be related to concentration effect due to inhomogeneous adsorption of anatyle or enhancement effect due to band-specific SERS characteristics or underneath nanoplasmonic characteristics. Two more possibilities which are related to the fluorescence background and the artifact of scanning probe techniques cannot be discarded as well^52,53^. To understand the reason behind, SERS spectral measurements were recorded at those specific regions of interest as marked by SP1 and SP3 in Fig. 3a,b. As shown in Fig. 3b, it was confirmed that the intensity was nearly one third lower compared to the intensity of the same band at hotsite (i.e. at the point SP4). However, the SERS band at 1572 cm^−1^ represents C–C stretching in phenyl ring that was not enhanced or activated at the hotspot defined by NP2–3–4–5. It is well-acknowledged that enhancement of specific SERS bands depends on various factors, viz. excitation wavelength, the molecular orientation, background emission and the interaction between the analyte and the plasmonic nanoaggregates.

It was reported by many groups, including Hayazawa and the team, that in near-field SERS measurements, it was possible to have some selective SERS bands that are not enhanced enough in far-field SERS measurements^49^. In fact, Hayazwa and the team reported some near-field SERS bands of R6G that were not even supported by DFT calculations accomplished by many groups, including Watanabe and the team^50^. At hotspots, near-field effect, such as evanescent fields, have a strong influence on near-field SERS measurements. In this study, some selective near-field SERS bands of R6G were observed that were not reported experimentally. However, these bands of R6G were well supported by DFT calculations done by Watanabe and the group^50^. Figure 5 displays near-field SERS mappings of these bands to demonstrate the correlation amongst strong SERS-active sites observed in individual SERS bands. Figure 5a–f show near-field SERS mappings of R6G extracted for the bands located at 1746, 1622, 1458, 1422, 832, and 649 cm^−1^ wavenumbers, respectively. The dashed squares, as mentioned in Fig. 5, guide the reader to follow the site of the strongest near-field SERS intensity for each individual band. It is noteworthy that the vertices of the four squares are pointing at the strongest sites, and all the strongest sites matched well to the interstitial “SP4” in the topography, as elaborated in the preceding section. It is well-known that the inherent nanogeometry of a particular interstitial determines how intense the localized EM near-field will be available to enhance the analyte laying at the same hotspot^12,18^.

### EM near-field distributions

There are two main mechanisms for SERS: the electromagnetic (EM) mechanism and the chemical (CM) mechanism^2,6,54^. The EM mechanism is based on the enhancement of the electromagnetic field induced by the localized surface plasmons of the metal nanostructures, which enhances the Raman signal of the analyte by several orders of magnitude. The CM mechanism, on the other hand, is based on the chemical interaction between the metal surface and the analyte, which results in a new Raman-active molecule or complex that exhibits a stronger Raman signal. The EM mechanism is the most prominent mechanism in SERS, and it is based on the excitation of surface plasmons, which are collective oscillations of the electrons in the metal nanostructures. When the plasmons are excited by the incident light, they generate a strong electromagnetic field that is highly localized around the metal nanostructures, leading to a substantial enhancement of the Raman signal of the analyte molecules in the near-field region. The CM mechanism, while less well-understood and less common, is often used to selectively enhance specific Raman modes of the analyte, overcoming some of the challenges associated with obtaining reproducible SERS measurements. Pitinger et al. hypothesized a two-fold EM enhancement mechanism as the fundamental mechanism of EM enhancement in the SERS process^55^. In the first phase, as defined in Eq. (1), EM near-field contributes to improving analyte Raman scattering, whereas the second process begins by improving scattered Raman light from adsorbed analytes. The details can be found elsewhere, and the references therein^56–58^. Therefore, the enhancement factor, M (including the first factor, M_1_, and the second factor, M_2_) for SERS is denoted by

1 $$M={\left|\frac{{E}_{L}({\lambda }_{I})}{{E}_{I}({\lambda }_{I})}\right|}^{2}\times {\left|\frac{{E}_{L}({{\lambda }_{I}\pm \lambda }_{R})}{{E}_{I}({{\lambda }_{I}\pm \lambda }_{R})}\right|}^{2}={{M}_{1}\left({\lambda }_{I}\right)\times M}_{2}\left({{\lambda }_{I}\pm \lambda }_{R}\right),$$

whereas ${E}_{I}$, ${E}_{L}$, ${\lambda }_{I}$, ${+\lambda }_{R}$ and ${-\lambda }_{R}$ are the incident electric fields, local electric fields, excitation wavelength, wavelengths of the anti-Stokes and Stokes Raman shifts respectively.

**Figure 6:**
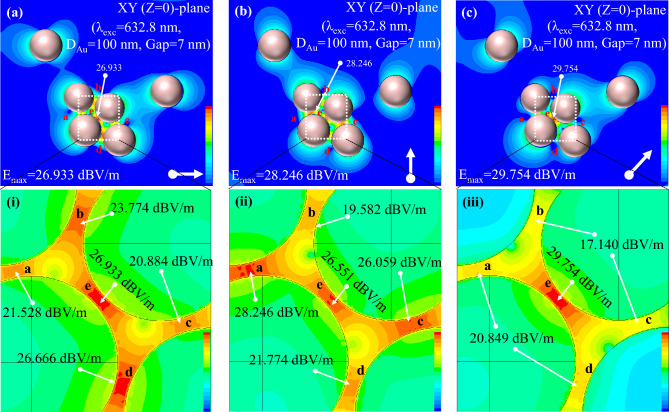
(**a–c**) EM near-field distribution at XY (Z = 0) plane of the similar gold aggregate model geometry used in FDTD simulation for excitation of 632.8 nm excited at s-, p-, and 45° of incident polarizations, respectively; inset (i)–(iii): Magnified portions of EM near-field distributions of the same model as marked in (**a–c**), respectively. The white arrows in each figure represent the polarization directions of incident excitation. Color bars represent the respective intensities observed in the simulations. Five interstitials of interest are marked as “1”, “2”, “3”, “4”, and “5” therein.

Since EM near-field distribution is the main ingredient in SERS enhancement, EM near-field distributions were extracted in this context for typical models as shown in Fig. 6. The model was designed and simulated for s-, p- and 45° of incident polarizations. Excitation of 632.8 nm that was normal to the geometry was used according to the experimental conditions. Five interstitials as marked by “a”, “b”, “c”, “d” and “e” were particularly highlighted and interestingly, these five interstitials corresponded to the interstitials as elaborated in Fig. 2a. The interstitials “a” and “c” represents the interstitials along the axis of 100° (between nanoparticles “2” and “3” and nanoparticles “4” and “5” respectively) whereas the interstitials “b” and “d” corresponds to those along the axis of 160° (between nanoparticles “2” and “4” and nanoparticles “3” and “5” respectively). And the interstitial “e” corresponds to the interstitial along the axis of 40° (between nanoparticles “2” and “4”). Figure 6a represents EM near-field distributions at XY (Z = 0) plane for a typical model geometry excited with incident excitation of s-polarization. Maximum EM near-field intensity of 26.933 dBV/m was found to be confined at the interstitial “e”, although the interparticle axis was out of plane to the incident excitation. A zoon-in view covering all the five interstitials was shown in inset (i) of Fig. 6a. It was noteworthy that the interstitials “e” and “d” were having almost similar intensities of EM near-field distribution (26.933 and 26.666 dBV/m respectively), although the interparticle axes were along ~ 40° and ~ 160° respectively. Maximum intensities of EM near-field distribution available at interstitials “a”, “b” and “c” were estimated to be 21.528, 23.774 and 20.884 dBV/m respectively as shown in inset (i) of Fig. 6a. The interparticle axes of interstitials “b” and “d” were found to be closer to s-polarized incident excitation compared to those of interstitials “a” and “c”. It has been reported that interstitial of interparticle axis parallel to incident polarization facilitates strong EM near-field localization^12,18^. This could be the plausible reason having strong EM near-field distribution at interstitial “d”. However, interestingly, the interstitial “e” was having the strongest EM near-field distribution at s-polarization of incident excitation, although the corresponding interparticle axis was far away, ~ 40° with reference to s-polarization. This indicated that the nanogeometry of interstitial is dominating over the influence of incident polarization in localizing EM near-field distribution. Under this circumstance, the interstitial having strong EM near-field distribution will provide strong enhancement in SERS measurements as observed herewith and demonstrated above.

In the case of p-polarization of incident excitation, it was expected that the interstitial of the interparticle axis parallel to incident excitation would support a strong EM near-field distribution. Figure 6b shows EM near-field distributions along the XY (Z = 0) plane of the same model excited with the p-polarization of incident excitation. As expected, the interstitials of the interparticle axis closely parallel to incident polarization were found to have strong EM near-field distributions. As stated earlier, the interparticle axes of interstitials “a” and “c” were found to be 100°, which is nearly parallel to the p-polarization of incident excitation. Therefore, the maximum intensities of EM near-field distributions at interstitials “a” and “c” were estimated to be 28.246 and 26.059 dBV/m, respectively, as shown in inset (i) of Fig. 6b. Inset (i) of Fig. 6b represents a zoom-in view of the selected area as marked by the white dashed square in Fig. 6b. As expected, the interstitials “b” and “d” had lower intensities of EM near-field distributions (E_max_ = 19.582 and 21.774 dBV/m, respectively) due to out of plane interparticle axes with reference to incident excitation. Interestingly, the interstitial “e” had a reasonably high intensity (E_max_ = 26.551 dBV/m) of EM near-field distribution similar to that occurring at s-polarization, although the corresponding interparticle axis was estimated to be ~ 40°. It was noteworthy that the interstitial “e” was more favorable in localizing EM near-field distribution regardless of incident excitation polarizations. In the case of oblique polarization (45°) of incident excitation, the interstitial “e” showed the strongest EM near-field distribution, as shown in Fig. 6c. Figure 6c displays EM near-field distributions along XY (Z = 0) plane of the same model excited with incident excitation of oblique (45°)-polarization. As expected, the interstitial “e” had the strongest EM near-field distribution (E_max_ = 29.754 dBV/m) due to its interparticle axis being closely parallel to the incident excitation polarization, as shown in inset (i) of Fig. 6c. Inset (i) of Fig. 6c depicts a zoom-in view of the selected area as marked by the white dashed square in Fig. 6c. It is to be noted that the intensities of interstitials “b” and “c” (E_max_ = 17.140 dBV/m) and those of interstitials “a” and “d” (E_max_ = 20.849 dBV/m) were found lower due to out-of-plane interparticle axes with reference to incident excitation of oblique (45°)-polarization.

For the convenience of simplicity and understanding the interparticle gaps of the FDTD model were kept 7 nm and the constituent nanoparticles were considered perfect spherical as shown in Fig. 6. However, nanoaggregate under this investigation was having difference interparticle gaps and constituent nanoparticles of different diameters as elaborated in Supporting Information. Regardless of the fact, it was noteworthy that the interstitial marked by “e” was exhibiting strong EM near-field localization at the incident polarization of 45° compared to other interstitials of the same FDTD model. Interestingly, the interstitial of the NP3 and NP4 that coincided well with this particular interstitial “e” of the FDTD model was observed to be a site of strong localized optical field (or hotsite) in near-field SERS measurements using a-NSOM setup as shown in Figs. 2 and 3. In addition, the EM near-field distribution the interstitials in FDTD simulation were noted to be drastically reduced with reference to the maximum EM near-field at interstitials as shown in Fig. 6. In this context, the near-field SERS intensities obtained at the sites marked by SP1, SP2 and SP5 as shown in Fig. 3 were recorded to be negligible with reference to that obtained at the hotsite.

## Conclusion

Near-field SERS spectroscopy of a well-defined gold nanoaggregate adsorbed with the Raman-active dye R6G was reported. A home-built a-NSOM setup facilitated recording spectrally- and spatially-resolved SERS measurements at the very same position without disturbing the specimen. A direct observation of a SERS-active hotspot was realized through near-field SERS measurements. A fairly good correlation between nanometric geometry and optical image was obtained through simultaneous measurements of shear-force topography and near-field SERS. The interstitial positioned at the center of the nanoaggregate provided the most intense Raman scattering, implying the possibility of a SERS hotspot therein. SERS bands of the spectrum of the Raman-active dye Rhodamine 6G recorded at the same hotspot coincided well with those reported so far. An FDTD model very similar to that under investigation was developed, and EM near-field distributions were extracted. A strong and localized EM near-field distribution at the interstitial positioned at the center of the tetramer was obtained, correlating well with the SERS hotspot observed in near-field SERS measurements.

### Supplementary Information

Supplementary Information.

## Data Availability

The datasets generated and/or analysed during the current study are not publicly available due to restriction from ongoing intellectual property application, but are available from the corresponding author on reasonable request.
