# Supplementary material for: Detection of surface enhanced Raman scattering active hotspot using near field scanning optical microscopy
Source: Sci Rep. 2024 May 8;14:10559. doi: 10.1038/s41598-024-61503-7 (PMC11078942; doi:10.1038/s41598-024-61503-7)
Supplement: Supplementary file 1 — Supplementary Information. [file 41598_2024_61503_MOESM1_ESM.pdf]

Supporting information

Detection of Surface Enhanced Raman Scattering Active Hotspot using Near Field Scanning Optical Microscopy

Mohammad Kamal Hossain[1,2,*]

[1]Interdisciplinary Research Center for Renewable Energy and Power Systems (IRC-REPS), Research Institute, King Fahd University of Petroleum & Minerals (KFUPM), Dhahran 31261, Saudi Arabia.

[2]K.A.CARE Energy Research & Innovation Center at Dhahran, King Fahd University of Petroleum & Minerals (KFUPM), Dhahran 31261, Saudi Arabia.

**Correspondence to: kamalhossain@kfupm.edu.sa*

1. Estimation of SERS enhancement factor:

To understand or quantify the number of dyes adsorbed on the SERS-active substrate is crucial to determine the enhancement factor. The group has elaborated the estimation of the dyes in this regard elsewhere [Phys. Chem. Chem. Phys., 2012, 14, 10132-10139]. However, in this current study, the enhancement factors were obtained following the revised formula,

$$EF = \left(\frac{I_{SERS}}{I_{bulk}}\right) \times \left(\frac{N_{bulk}}{N_{SERS}}\right),$$

whereas the ratio between N_{bulk} and N_{SERS} is equal to the ratio between C_{bulk} and C_{SERS} (i.e. $\frac{N_{bulk}}{N_{SERS}} = \frac{C_{bulk}}{C_{SERS}}$). The detailed calculation can be shown as follows.

As per the abovementioned reference, the number of analytes in bulk condition was calculated according to the following formula

$$N_{bulk} = \frac{C_{bulk} \times V_{bulk} \times N_A}{A_{drop} \times D_{laser}} \qquad \text{(eq. 1)}$$

where N_{bulk}, C_{bulk}, V_{bulk}, N_A, A_{drop} and D_{laser} represent the number of analytes, concentration of dye, volume of dye solution, Avogadro number, area of the droplet in incubation and laser spot diameter in Raman measurements respectively.

The number of analytes in the SERS condition was calculated according to the following formula

$$N_{SERS} = \frac{C_{SERS} \times V_{SERS} \times N_A}{A_{drop} \times D_{laser}} \qquad \text{(eq. 2)}$$

where N_{SERS}, C_{SERS}, V_{SERS}, N_A, A_{drop} and D_{laser} represent the number of analytes, concentration of dye, volume of dye solution, Avogadro number, area of the droplet in incubation and laser spot diameter in SERS measurements respectively.

In this current study, the volume of dye solution and laser spot diameter remained the same in all the Raman and SERS measurements. The areas of the droplet in incubation in both cases will be the same because of the same volume of dye solution. Therefore, solving the eq. 1 and eq. 2, one can notice, the ratio between the number of dyes adsorbed on nanoaggregate and that at bulk condition is,

$$\frac{N_{bulk}}{N_{SERS}} = \frac{C_{bulk}}{C_{SERS}}$$

Considering the concentrations of SERS condition (1×10^{-6} M of R6G) and bulk (0.2 M of R6G) condition, one could note that the estimated enhancement factor reached in the range of 10^{6}.

2. **Estimation of interparticle gaps:**

The nanoaggregate used under this study was comprised of four spherical nanopartilces as marked by NP2, NP3, NP4 and NP5 in Fig. 2a (main draft) and Fig. SI1 (in SI). The near-field SERS measurements using a-NSOM set-up confirmed that the interstitial between NP3 and NP4 was turned into hotsite and strong correlated SERS fingerprints of R6G was recorded. However, it is well-acknowledged that the interparticle gap play crucial role in hotsite generation. Fig. SI1 shows the shear-force topography of a-NSOM measurement captured simultaneously to the near-field SERS measurement. Within the naoaggreate five interestitials were noted and interparticle gaps of such fine interstitials were estimated using image processing tool, ImageJ. ImageJ is a Java-based image processing program developed at the National Institutes of Health and the Laboratory for Optical and Computational Instrumentation. The interparticel gaps between NP2 and NP3, NP3 and NP5, NP5 and NP4, NP4 and NP2 and NP3 and NP4 were estimated to be 103.4, 106.7, 98.6, 98.5 and 107.8 nm respectively. Considering the deviation in diameters of two nanospheres in the

range of ~10%, the interparticle gap lies within the range of 0 nm to 7.8 nm. However, we acknowledge that a direct measurement of interparticle distance between two interacting nanoparticles in the nanoaggregate is limited by the a-NSOM tip convolution.

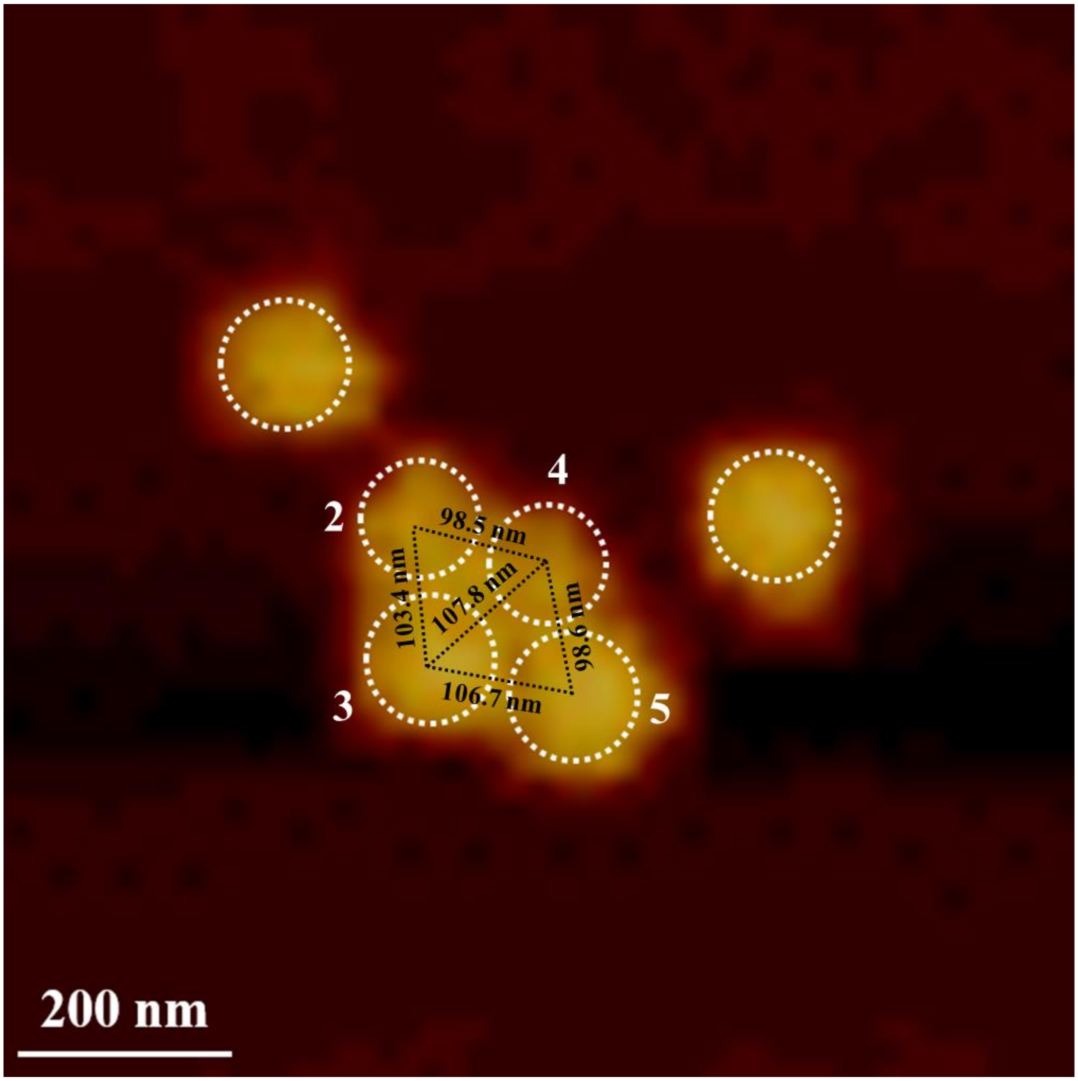

Fig. SI1: Shear-force topography of a-NSOM measurement captured simultaneously to the near-field SERS measurements.
